# Insulin secretion from beta cells within intact islets: Location matters

**DOI:** 10.1111/1440-1681.12368

**Published:** 2015-03-27

**Authors:** Oanh Hoang Do, Peter Thorn

**Affiliations:** ^1^ School of Biomedical Sciences University of Queensland St Lucia Brisbane Qld Australia

**Keywords:** beta cells, calcium, diabetes, exocytosis, insulin, islets, synapse

## Abstract

The control of hormone secretion is central to body homeostasis, and its dysfunction is important in many diseases. The key cellular steps that lead to hormone secretion have been identified, and the stimulus‐secretion pathway is understood in outline for many endocrine cells. In the case of insulin secretion from pancreatic beta cells, this pathway involves the uptake of glucose, cell depolarization, calcium entry, and the triggering of the fusion of insulin‐containing granules with the cell membrane. The wealth of information on the control of insulin secretion has largely been obtained from isolated single‐cell studies. However, physiologically, beta cells exist within the islets of Langerhans, with structural and functional specializations that are not preserved in single‐cell cultures. This review focuses on recent work that is revealing distinct aspects of insulin secretion from beta cells within the islet.

## Introduction

Understanding the control of hormone secretion is of central importance to furthering our knowledge of body function and in preventing and curing disease.^1^ Over the past 40 years, there has been dramatic progress in our knowledge, and at the cellular level, the essential outline of the stimulus‐secretion pathways for many endocrine cells is understood.^2^ However, in the body, endocrine cells are often tightly packed within endocrine glands. The secretory output from a gland can be very different from isolated single cells,^3^ with factors such as gap junctional links and paracrine effects strongly influencing the control of secretion.^4^, ^5^ The ideal method for determining how endocrine cells really behave would measure secretion from single cells within intact endocrine glands in a living animal. This has yet to be achieved, but progress is being made, and this review will focus on some recent findings that improve understanding insulin secretion from beta cells within islets of Langerhans.

## Insulin secretion *in situ* within intact islets

Insulin secretion is an integral component in the control of blood sugar levels. Insulin is produced in pancreatic beta cells and is packaged into membrane‐bound secretory granules, with thousands of granules present in each cell. Stimulation of beta cells by glucose or other secretagogues leads to the fusion of a small number of these granules with the cell membrane and to the release of insulin to the outside of the cell.^6^, ^7^ At the cellular level the stimulus‐secretion pathway for glucose is well understood and is dependent on an influx of calcium through voltage‐sensitive calcium channels.^8^ Other secretagogues, such as glucagon‐like peptide‐1, act through cyclic adenosine monophosphate to augment secretion.^9^ Ongoing work is defining the key molecular players in these stimulus‐secretion coupling pathways and building up a picture of secretory control.

Most of this knowledge of the control of insulin secretion has been obtained from beta‐cell lines and isolated, cultured single beta cells. However, it is well known that isolated beta cells behave differently than beta cells within intact islets.^3^, ^4^ If we focus on glucose‐induced insulin secretion, for example, it is known that single cells have elevated basal levels of insulin secretion and a blunted maximal insulin secretory response to glucose. This leads to a ‘compressed’ glucose dose‐response relationship in isolated cells compared to that in intact islets.^3^, ^10^ The possible factors that can explain these differences include beta‐cell‐to‐beta‐cell interactions, interactions between the beta cells and the vasculature, and interactions among the different cell types within the islet.

### Beta‐cell‐to‐beta‐cell interactions

The endocrine cells within the islets of Langerhans are tightly packed together and well supplied with blood vessels.^11^, ^12^ In the rodent islet, beta cells are grouped together in the core of the islet, and the other types of endocrine cells are around the periphery. In human islets, the endocrine cells are interspersed, but the major cell type in any healthy islet are the beta cells.^13^ Therefore, in both rodent and human islets, beta cells are in contact with other beta cells, and these contact areas are likely to occupy the majority of the membrane surface area of each beta cell.

**Figure 1:**
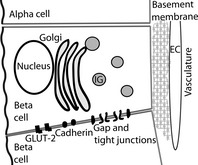
A diagram emphasizing the spatial relationships of beta cells to their surrounds within the islet**.** Beta cells make homotypic contacts with adjacent beta cells through cadherins, gaps, and tight junctions. The region of beta‐cell‐to‐beta‐cell contact is also enriched in the glucose transporter (GLUT‐2). Beta cells may relate to other types of endocrine cell, such as alpha cells, through paracrine or whole body communication. Finally, beta cells sense the basement membrane along the vasculature, probably through integrin receptors. EC, endothelial cell; IG, insulin granule.

Electron microscopy shows the membrane areas of beta‐cell‐to‐beta‐cell contact contain tight junctions and gap junctions that appear to be arranged in discrete patches.^14^ In addition, cadherin junctions are present along the beta‐cell‐to‐beta‐cell membrane contact areas (Fig. 1).^15^ In terms of function, the gap junctions are the best studied, and these play a major role in coordinating electrical activity across the islet.^4^ This in turn coordinates the calcium responses and is therefore likely to couple the secretory output of the beta cells, although this has not directly been shown. In isolated single cells, increasing glucose concentrations leads to increasing recruitment in the numbers of cells that respond, suggesting beta‐cell heterogeneity in sensitivity to glucose.^16^ Gap junctional links in islets would coordinate cell responses and tend to work against this heterogeneity. It would be predicted that at low, threshold glucose levels, a majority of non‐responding cells in an islet would dampen the activity of any sensitive, responding cells. In contrast, as the glucose concentration is increased, an increasing recruitment of responses from beta cells would tend, through the gap junctional links, to increase the activity of neighbouring non‐responding cells. The overall effect would be to stretch the glucose dose response in the islet compared to single cells.^17^ Support for this hypothesis comes from experiments using connexin 36 knockout animals, although the picture appears more complex with other additional factors also coming into play in the islet.^10^, ^18^

### Beta‐cell‐to‐vasculature interactions

Islets of Langerhans are richly vascularized, and measurements suggest that most beta cells have one or more points of contact with the blood vessels of the capillary bed.^12^, ^19^ Developmentally, it is vascular endothelial growth factor A secretion from the endocrine cells that attracts incoming endothelial cells into the growing islet.^20^ In turn, the endothelial cells secrete basement membrane, which is used by the beta cells as cues for their growth and possibly orientation and function.^20^, ^21^ These actions of the basement membrane are not well understood *in vivo*, but there are a number of studies that have shown that the survival and secretory output of isolated cells can be manipulated by adjusting factors that are present in the basement membrane.^22^, ^23^ We conclude that while the importance of beta‐cell‐to‐vascular interactions is not yet well understood, it is likely to be a key component in understanding beta‐cell function within islets.

### Beta‐cell‐to‐endocrine‐cell interactions

Although glucose is a main driver for insulin secretion, beta cells also integrate a variety of other stimuli, such as hormones and neuronal inputs, to finely control insulin secretion. One possible source of stimulation comes from the different endocrine cells within the islet, which might communicate in a local, paracrine manner. In support of this idea, somatostatin knockout animals show increased insulin secretion in response to glucose and pancreatic beta cells, which have been shown to influence glucagon secretion.^5^, ^24^, ^25^

However, the central problem is that although actions of substances secreted from one endocrine cell may be shown *in vitro* to have actions on other endocrine cells, this does not prove paracrine action. In a simple but powerful experiment, Kawai *et al*.^26^ showed that the concentrations of somatostatin and glucagon in the venous outflow of the pancreas were much higher than necessary to produce effects on the islet when injected into the arteriole pancreatic supply. The implication is that somatostatin and glucagon are secreted into the blood stream, away from the islet, at concentrations that are too high for them to have any local physiological effect on the beta cells. These experiments therefore suggest that the pancreatic beta cells are not responding to locally released somatostatin and glucagon.

In addition to paracrine actions on the beta cells, there is also the possibility of an autocrine action of insulin to feedback onto the beta cells. On the basis of mathematical modelling, an argument has been made that the insulin concentrations in the vicinity of secreting beta cells would be too high to explain the known insulin actions on beta cells.^27^ This supports the idea that beta cells might preferentially secrete insulin into the blood stream where it would be carried away from the islet. As pointed out by Kawai *et al*.,^26^ this implies that within single beta cells there is a segregation of plasma membrane domains into regions where secretion occurs and regions where the hormone receptors are located. In support of the idea that beta cells have functionally segregated membrane domains, the glucose transporter 2 (GLUT‐2) transporter, the primary route of glucose entry into rodent beta cells, is exclusively located on the lateral membranes that lie between beta cells.^28^ We conclude that while in principle beta cells can respond to local paracrine and autocrine influences, more work is needed to determine if this occurs *in vivo*.

### *In situ* measurement of insulin secretion in intact islets

Insulin secretion from intact islets is usually based on work measuring the total islet insulin secreted using an insulin assay.^3^ However, this method cannot determine how each individual beta cell responds, and given the complexities within the islet, we can imagine many different scenarios for beta‐cell recruitment and response. For example, the dose‐dependence of glucose‐induced insulin secretion could arise due to either the progressive recruitment of more beta cells to respond or all beta cells responding and dose‐dependence increasing secretion from each beta cell. To test these hypotheses, we cannot use single‐cell methods of measuring secretion such as total internal reflection fluorescence microscopy or capacitance recording. An alternative method that can simultaneously measure secretory responses from a number of cells is the use of two‐photon microscopy, which has been employed as an assay for granule fusion in the islets and other tissues.^29^, ^30^

**Figure 2:**
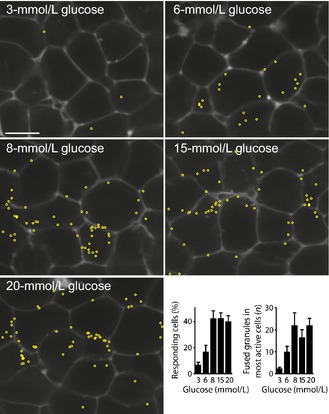
Live‐cell two‐photon recordings show the glucose dose‐dependence of insulin granule fusion. Two‐photon microscopy was used to record the entry of extracellular dye (seen outlining the cells) into individual insulin granules as they fuse with the cell membrane over 20 min in response to glucose addition. Each granule fusion event was identified as the sudden appearance of a bright spot of fluorescence ~300 nm in diameter, and its position is marked by a yellow circle. The number of fusion events increased in a dose‐dependent manner which could be quantified both as an increase in the numbers of responding cells and an increase in the number of fusion events per responsive cell. Figure modified from Low *et al*.^31^

Two‐photon pulsed laser excitation only excites within a shallow depth above and below the plane of focus. In the granule fusion assay, the islets are bathed in fluorescent dye, and because the cells exclude the dye, they appear as dark regions surrounded by intense extracellular fluorescence (Fig. 2). When a secretory granule fuses with the cell membrane, the extracellular dye enters through the fusion pore and the granule appears as a bright spot of fluorescence on the edge of the cell.

As with any method, this two‐photon assay has its limitations. One criticism is that it is a measure of endocytosis, and any uptake of dye could be considered to be endocytic. However, we and others use the kinetics of the fluorescent signal as an indication that we are studying the time immediately after granule fusion rather any later period of endocytosis.^29^, ^31^, ^32^ The rapid appearance of the fluorescence signal, with rise times of less than 2 s and that are dependent on the size of the extracellular fluorescent probe (the larger probes have slower rise times),^29^ argues strongly that we are studying the flow of dye into the granule immediately after the opening of a fusion pore, rather than the uptake of dye through the endocytic engulfing of an extracellular volume. Another criticism is that the extracellular dye will label any exocytic process and is not specific to beta‐cell insulin secretion. To address this, we have performed a range of control experiments that are all consistent with insulin granule fusion. These experiments show consistency with the size of the cells (beta cells are bigger than other endocrine cells); the size of the fluorescently labelled structures, which are consistent with insulin granules; immunostaining of insulin within granules that have taken up the extracellular dye; and consistent glucose dose‐dependence of the frequency of fusion events with the expected amounts of insulin secretion.^31^

We conclude that the two‐photon method is an interesting additional technique that has specific, and currently unique, applicability in determining the single‐cell insulin secretory responses within the islet. The principal advantage of the two‐photon method is the ability to record across a number of cells within the islet. We have shown that at low glucose concentrations (< 6 mmol/L), there are numerous apparently non‐responding cells.^31^ This is consistent with the heterogeneity in beta‐cell responses observed in single‐cell studies.^16^ At higher glucose concentrations, we show more cells are recruited to respond, and the number of insulin granule fusion events within each cell increases.^31^ This suggests that cell responses are coordinated, probably by gap junctional communication.^10^ Further evidence that supports a coordinating role of gap junctions is that granule fusion events are coordinated in time across a number of cells and occur as waves of activity.^31^ This is consistent with the waves of calcium signals that pass from cell to cell and are coordinated by gap junctions.^33^ However, our data show that the most significant factor in glucose dose‐dependence is not the increased recruitment of cells (which shows approximately a four‐fold increase) but rather the increase in the number of granule fusion events per cell (which shows approximately a nine‐fold increase). This indicates that the intracellular stimulus‐secretion coupling pathway is the main driver for secretion.^31^ We note here that, in addition to calcium, there are many other factors that drive glucose‐induced secretion within cells,^34^ such as mitochondrial activation.^35^ Our work highlights the importance of these drivers in the overall dose‐dependent actions of glucose on insulin secretion from islets.

The two‐photon method can also give insights into the behaviour of granules during fusion. It is known that after fusion, the fusion pore can either dilate and the secretory granules collapse into the cell membrane or the fusion pore can close. This latter pore closure can be followed either by a reopening of the pore or a recovery of the granule in a process commonly called kiss‐and‐run exocytosis. Reports by Tsuboi and Rutter and by MacDonald *et al*.^36^, ^38^ have provided good evidence that insulin granules can show fusion pore dynamics and kiss‐and‐run exocytosis. These fusion pore dynamics might be important in regulating the types of substances released from the granule and the kinetics of that release.^38^ However, direct observation in intact islets indicates that kiss‐and‐run granule fusion is rare.^29^, ^39^ Indeed, if kiss‐and‐run was a significant mode of exocytosis, then this would be observed by the two‐photon technique as a gradual accumulation of fluorescently labelled granules – something that is not seen.^29^, ^31^ Our recent data use a granule acidification assay that records the time point of granule fusion (as the entry of extracellular dye) and then determines if the granule subsequently acidifies, an indication that the fusion pore has closed; the vascular‐type H^+^‐ATPase then acidifies the granule lumen. We show that granule acidification is observed, confirming that kiss‐and‐run does occur. However, it is seen in less than 10% of all fusion events, and this proportion does not change across the glucose dose range.^31^ We conclude that neither kiss‐and‐run nor granule fusion pore closure (a requisite for kiss‐and‐run) is common in beta cells. However, because the granule acidification method we employ has a relatively slow temporal resolution that is dependent on the action of the vascular‐type H^+^‐ATPase, we do not exclude the possibility that the fusion pore could open and close quickly. Determination of more rapid opening and closing of the fusion pore behaviour requires modifications to the two‐photon method or, ideally, capacitance measurements.^40^ These methods have not been performed in islets, but pore behaviour remains an important issue because it could be a major factor in controlling the release of granule content.

Another granule behaviour that is readily observed with two‐photon microscopy is compound exocytosis.^41^ Multiple granule fusion events can be observed occurring at the same position on the cell membrane.^32^, ^41^ This suggests that granules are fusing with each other in a sequential manner, although additional methods are really needed to prove granule‐to‐granule fusion rather than granules fusing with the cell membrane and being very close to each other.^42^ There is also some evidence that granules fuse with each other within the cell; in this case, the final fusion event with the cell membrane would be observed as a larger object than a single insulin granule.^32^

### Conclusions

We conclude that the beta‐cell environment within intact islets is complex and can lead to many factors that might alter cellular behaviour. Increasing the glucose concentration leads to the recruitment of beta cells, supporting the idea of heterogeneity in cell sensitivity to glucose, and to an increase in the numbers of fusing granules within each beta cell. The beta‐cell responses are coordinated in time, which emphasizes the importance of cell‐to‐cell coupling. Within an islet, the potential functional role of beta‐cell interactions with the vasculature and other cell types remains to be fully resolved.

## Studying beta‐cell secretory dysfunction *in situ*

The role of beta‐cell dysfunction in the onset and progression of diabetes mellitus type 2 is of great importance. Impaired beta‐cell function is one of the earliest detectable defects in first relatives of diabetes mellitus type 2 patients.^2^, ^43^, ^44^, ^45^

Lepr^db^ (db/db) mice are a long‐standing and extensively used model for diabetes mellitus type 2. The mice have a spontaneous mutation on chromosome 4 that leads to leptin receptor deficiency. The mice gain weight and develop the characteristics of the disease after about 4 weeks of age; disease progression in the mice follows human diabetes mellitus type 2 and includes obesity, insulin resistance, hyperinsulinaemia, and finally hypoinsulinaemia and hyperglycaemia.^46^, ^47^, ^48^

Previous studies that measured insulin secretion from islets have suggested a significant impairment in islet insulin secretion of db/db islets.^49^, ^50^, ^51^ Studies have identified changes in the expression and behaviour of components of the stimulus secretion pathway, but a unified model determining the relative importance of these changes has not been determined. In particular, how these changes are manifest within intact islets is unknown. Here, we will focus on the db/db model and discuss the known cellular changes using the framework of the stimulus‐secretion cascade, which starts with the uptake of glucose into the cell, then glucose metabolism, an increase in the adenosine triphosphate/adenosine diphosphate ratio, closure of the adenosine triphosphate‐dependent potassium channels, influx of calcium, and finally fusion of insulin‐containing granules to the plasma membrane.^52^, ^53^, ^54^

### Cellular changes: defects in glucose uptake and glucose metabolism

In db/db mice, there is evidence of decreased expression of GLUT‐2 and decreased adenosine triphosphate production.^55^ Clearly, this would affect all the downstream signalling pathways. It is not clear by how much expression of GLUT‐2 protein decreases, but measures of messenger RNA show ~50% reduction.^56^ What is interesting is that GLUT‐2 expression can rapidly recover (within 2 weeks) after manoeuvres to normalize glucose levels.^55^, ^56^ This suggests its expression is labile and highly sensitive to the sugar levels of the environment.

### Cellular changes: defects in calcium signalling

The normal cellular calcium response to glucose is dominated by calcium influx through voltage‐sensitive calcium channels. In the db/db model, the cytosolic calcium response to glucose rises more slowly than in controls and reaches a lower maximum level.^51^, ^57^, ^58^ Furthermore, it appears that the oscillations in calcium, which are seen in controls, are altered in the db/db responses.^51^, ^57^ All of these parameters – the slower rise, the reduced maximum, and the changes in oscillations – are likely to be critical determinants of the final secretory output. It is therefore essential to quantitatively analyse these responses. Studies that calibrate the Fura‐2 signals from intact islets, including our own,^59^ determine the absolute values of cytosolic calcium in response to glucose. Our results show that the peak calcium response is significantly smaller (281 *vs* 193 nM) in the db/db than in the wild‐type islets; these results are similar to those of Gustavsson *et al*.^58^ Both studies also highlight another difference and show a significant elevation in resting calcium concentrations (in our study 37.5 *vs* 56.8 nM) in the db/db islet compared to the wild‐type islet,^58^, ^59^ which may explain the elevated tonic secretion of insulin that is observed in db/db islets.^56^, ^57^, ^59^

In addition to these defects in calcium signalling in the db/db islet, there also appears to be a downstream defect in the beta cell's ability to respond to a calcium rise. In our study, we showed this with ionomycin, a calcium ionophore that bypasses all the steps prior to calcium entry and that increased insulin secretion in wild‐type (by about 60% above basal) but not in db/db islets.^59^ This indicates a defect in the later stages of the secretory cascade such as the control of granule delivery to the plasma membrane or subsequent granule fusion. This idea is also supported by work in other models of diabetes.^60^, ^61^

### Cellular changes: defects in granule fusion

There is evidence from single‐cell studies that granule fusion can be complex, with the fusion pore opening and closing and the granules undergoing kiss‐and‐run exocytosis. There are two reports that have studied this behaviour in models of diabetes but none in the db/db model. In the first, Tsuboi *et al*. measured full fusion (where all granule content is lost) and partial fusion (where only some granule content is lost) using labelled granule content proteins in cultured rat pancreatic beta cells.^62^ They showed that after culturing in 30‐mmol/L glucose for 48 h (a model for glucotoxicity), there was a selective decrease in full fusion exocytosis from 25% of all the exocytic events to 5%.^62^ This idea that full fusion is specifically decreased is supported by work on the Goto‐Kakizaki rat model of diabetes.^63^ Here, changes were shown in the apparent behaviour of the granules prior to fusion (docked *vs* newcomers) with an overall reduction in granule fusion.^63^ The single‐cell measurements point to a defect in granule fusion, but from this work, it is unclear how beta cells within a disease islet might be behaving. Using similar arguments to those used for normal islets, we could imagine a number of scenarios to describe what happens in disease. An overall reduction in islet secretion might therefore be due to complete loss of secretion from a small number of cells or a partial loss from all beta cells. This is becoming an important issue because we now understand that beta‐cell phenotype changes in disease and leads to cellular dedifferentiation.^64^ Understanding the cellular secretory phenotype would therefore give insights into disease progression.

Our experiments used the advantages of the two‐photon methods to record single insulin granule fusion events within optical cross‐sections of ~20 cells within intact islets.^59^ Analysis showed that the total number of insulin granule fusion events, induced by 15‐mmol/L glucose, was decreased by 83%. This overall decrease is a reflection of a 73% reduction in the number of responding cells and a 50% reduction in the number of granule fusion events per responsive cell.^59^ Clearly, the prevalent factor is the loss of cells that are recruited to respond, and this could be due to dedifferentiation of the beta cells in the diabetic environment.^64^

We also set out to determine the characteristics of granule fusion in the diseased islets from db/db mice. Our study shows a wide spectrum of fusion events with different postfusion behaviours (granular lifetime and postfusion fluorescent intensity) in both control and db/db islets.^59^ However, we observed a similar pattern of frequency distributions in both granular lifetime and postfusion fluorescent intensity in wild‐type and db/db islets, indicating these are not factors that change in disease. Interestingly, by combining a pH‐insensitive (sulforhodamine B) and a pH‐sensitive dye (8 hydroxypyrene‐1,3,6 trisulfonic acid), we observed a higher rate of recaptured granules or kiss‐and‐run events in db/db islets compared to the wild‐type islets (11.6 *vs* 6.6%, *P *= 0.02). However, the reduction in fusion granules undergoing full fusion was large and was sufficient to explain the impaired insulin secretion in db/db islets. We conclude that, at least in this diabetic model, the differences in the kinetics of insulin granules do not play a significant role in the decreased hormone output.

### Cellular changes: changes in granule fusion proteins

The evidence points to at least one component of disease being a loss of full fusion, and because granule fusion is dependent on soluble *N*‐ethylmaleimide‐sensitive factor attachment proteins, these have been thought of as candidates that might underlie the secretory changes seen in the diabetic islet.^65^ In the db/db model, recent work using quantitative polymerase chain reaction showed that synaptosomal‐associated protein 25 and vesicle‐associated membrane protein 2 (synaptobrevin) expression was upregulated and syntaxin‐1A expression was downregulated.^48^ We looked directly at protein expression by comparing the immunostaining of wild‐type and db/db islets; our results showed a similar profile of *N*‐ethylmaleimide sensitive factor attachment protein changes with ~50% increase in synaptosomal‐associated protein 25 and vesicle‐associated membrane protein 2 and ~35% decrease in syntaxin‐1A in db/db islets.^59^ This reduction in syntaxin‐1A could contribute to the secretory defect as it is one of the three main components of the fusion *N*‐ethylmaleimide sensitive factor attachment protein complex.

### Conclusions

We conclude that recording beta‐cell behaviour within an intact islet can reveal the relative importance of the various factors that are thought to play a role in disease. Our current understanding from this work is that the loss of insulin secretion, observed in diabetes mellitus type 2, can largely be accounted for by a reduction in the numbers of responsive cells. This might arise as a result of dedifferentiation of beta cells. In addition, studies have shown specific defects in the components of the beta‐cell stimulus‐secretion cascade, although the relative contribution of each component to disease is not known.

## Targeting of insulin secretion to the blood

We have already discussed the control of insulin granule fusion in single beta cells within healthy and diseased islets. We focused firstly on counting the number of granule fusion events as a direct measure of insulin secretion and secondly on the dynamics of granule fusion. However, there is an additional aspect of granule fusion that could be important: the spatial distribution across the beta cell.

As discussed previously, there is evidence for distinct membrane domains in beta cells,^28^, ^66^ with a lateral domain between the beta cells containing the GLUT‐2 transporter, which suggests that this is another domain because it is excluded from the vascular face of the beta cells. The distribution of insulin granule fusion is not well understood, but we might imagine three distinct models for the secretion of insulin from individual beta cells: (i) insulin might be secreted all around the cells; (ii) it might be secreted exclusively into the lateral domain; or (iii) it might be secreted into the vascular domain. The spatial differences in these three models would lead to very different consequences in terms of autocrine and paracrine actions of insulin within the islet. In addition, within the cell, local targeting of secretion would require specialized machinery in the stimulus‐secretion cascade.

Here, we discuss evidence that, in the native islets of Langerhans, insulin secretion is targeted towards the vasculature at a specialized region of the cells we have termed the ‘endocrine synapse’.

### Indirect evidence for targeted secretion in beta cells

The main methods that have been used to measure insulin secretion are centred on single‐cell techniques. Here, single‐cell calcium responses to glucose appear to be spatially asymmetric,^67^, ^68^ and indirect staining (quinacrine) suggests that the insulin granules may be clustered at the same region.^68^ Further evidence for spatial asymmetry in the secretory response was obtained from the use of carbon fibre electrodes and showed that insulin secretion is not evenly distributed across the cell membrane.^69^ This result is directly contradicted by the work of Rutter *et al*.,^70^ which showed that granule fusion (as assessed with a granule‐specific fluorescent probe) occurs randomly across the beta‐cell membrane. One problem with these experiments is that they are based on 2‐D cell cultures, which will not replicate the 3‐D environment of the islet; it is therefore possible that experimental differences in the cell culture could be influential in beta‐cell orientation and may explain the contradictions between the data. Whatever the explanation, this work on isolated cells is not readily applicable to what might be happening in the much more complex environment of the islet. Work in intact islets from Bonner‐Weir concluded that insulin secretion was likely to be targeted towards the vasculature.^19^ This idea was based on electron microscopy, where chronically stimulating the cells caused a substantial depletion of the numbers of insulin granules; when imaged, the remaining granules showed a preferential distribution towards the vasculature.^19^

### Direct measurements of secretion

**Figure 3:**
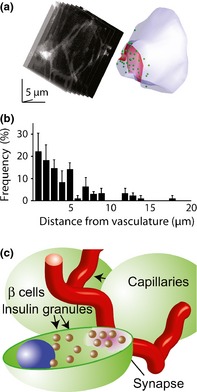
Live‐cell 3‐D two‐photon recording of a single beta cell within an islet shows targeting of insulin granule fusion to the vasculature. (a) The basement membrane of the vasculature was stained with isolectin B4 and is shown on the reconstructed cell diagram as the red area. Sequential Z planes were recorded through the cell during a response to stimulation, and each granule fusion event is identified as a green circle on the reconstructed cell. (b) The location of each granule fusion event for a number of cells was measured relative to the isolectin B4 staining and, when plotted out, shows a strong bias towards the vasculature. Figure modified from Low *et al*.^71^ (c) Diagram of the proposed synaptic connection at the beta‐cell membrane that adjoins the vasculature.

The live‐cell two‐photon assay should lend itself to addressing the issue of targeted secretion of insulin. Indeed, as a side observation in a paper focussed on granule kinetics, Takahashi *et al*.^29^ group suggested that insulin granule fusion occurred predominantly away from the vasculature. However, here it is important to understand that the two‐photon assay provides an optical cross‐section through an islet that is around 1–2 *μ*m thick. In this way, XYT image sequences, as used in this study, are not a good determinant for the 3‐D structure of an islet. So, if a blood vessel is actually imaged within the two‐photon optical section, then the closeness of granule fusion events in the adjacent cells can be measured. However, cells in the same two‐photon section that appear to be distant from blood vessels could actually be close to other blood vessels that lie just outside the two‐photon section. The analysis by Takahashi *et al*.^29^ includes all cells in all sections and therefore does not account for this problem. In our initial experiments, we also performed XYT imaging, but in our analysis, we only included those cells that were adjacent to blood vessels, which led us to show a bias for granule fusion events occurring close to the vasculature.^71^ However, our most convincing data for targeted secretion come from work using 3‐D two‐photon imaging. This enabled us to record single granule fusion events over time from multiple Z sections across single beta cells within intact islets.^71^ The data strongly support the idea that granule fusion is directed towards the vasculature (Fig. 3).

### Molecular mechanism that target insulin secretion to the vasculature

This work, indicating that insulin secretion is targeted towards the vasculature, suggests that there are molecular mechanisms that must control this targeting of granule fusion. We know that an analogous targeting of secretion occurs in neurones, where neurotransmitter release is restricted to the presynapse and is supported by presynaptic molecular machinery.^72^ In beta cells, evidence shows that some of these presynaptic proteins are present and that they are functionally important for insulin secretion. For example, piccolo and Rab3‐interacting molecule 2a have functional effects that suggest a role in insulin secretion, although their spatial distribution is unknown.^73^, ^74^ There is one paper that analyses the beta‐cell location and function of another presynaptic protein, protein rich in the amino acids E, L, K, and S (ELKS), which is a scaffold protein that interacts with other synaptic proteins and supports the targeting of neurotransmitter release at the synapse.^72^ In the beta cells, ELKS is enriched in the membrane close to the blood vessels and is involved in secretory control.^75^ However, the 3‐D distribution of ELKS was not determined, and furthermore, if we are to show that there exists a presynaptic‐like complex in beta cells, it is important to determine the relative location of other potential protein partners that make up the complex.

To this end, we have employed 3‐D immunofluorescence, which enables us to study the distribution of these proteins in the context of the complex 3‐D structure of the islet. Furthermore, we have employed the pancreatic slice technique, pioneered by Rose *et al*.^60^, which enables us to obtain tissue within a few minutes of killing the animals. This is important because it is well known that the more usual enzyme‐dependent islet isolation method leads to tissue damage.^12^ Using this slice method, we confirmed that ELKS is specifically located at the vascular face of the beta cells, and to extend these findings, we looked at other synaptic proteins and showed that Rab3‐interacting molecule 2a, liprin‐*α*1, and piccolo are all present on the same vascular face of beta cells.^71^

### Conclusions: a model for targeted secretion

These data imply that a ‘presynaptic’ specialized region exists on the vascular face of beta cells, which we have termed the ‘endocrine synapse’ (Fig. 3c). If this is true, then what is its functional significance for the control insulin secretion?

The most obvious implication is that there may be a spatial segregation of functions in the different plasma membrane domains of the beta cell. The lateral membrane domain, between adjacent beta cells, contains specialized junctional proteins and is enriched in the GLUT‐2 transporter. It would thus appear that the lateral membrane is distinct from the membrane domain of the vascular face of the beta cell, which is associated with the enrichment of the synaptic proteins, such as ELKS and liprin. Functionally, insulin secretion targeted to the blood would be rapidly transported away from the islet and limit any paracrine actions of local and, therefore, high concentrations of insulin.

In addition, targeting insulin secretion would be likely to affect the intracellular secretory control cascade. Based on the currently identified components, the synaptic region would be capable of localizing insulin granules at the vascular face, through Rab3‐interacting molecule 2a, ELKS, liprin, and piccolo interactions. This alone may be sufficient to target secretion. However, a further crucial factor in secretory control is the relative position of calcium channels to the exocytic machinery, with biophysical evidence indicating that calcium influx through a few channels is required to induce exocytosis.^76^ We therefore might speculate that calcium channels are additional components at the synapse.

Finally, there must be cues that the cell is using to orientate the synaptic machinery to the vascular face of the beta cells. Neurons use neuroligin‐neurexin extracellular interactions to orientate presynaptic and postsynaptic regions;^77^ both proteins have been found in islets and may form links between the beta cells and endothelial cells.^78^ Another possibility is that beta cells sense the vascular basement membrane, an interaction already known to have functional consequences.^21^

## Conclusions

The pancreatic beta cell is one of the best studied secretory cell types. Ongoing work is now placing the beta cell in the context of its native environment of the islet. This is revealing how insulin secretion is controlled physiologically and what goes wrong with secretion in disease.
